# A new approach in the one-step synthesis of α-MnO_2_*via* a modified solution combustion procedure

**DOI:** 10.1039/d2na00257d

**Published:** 2022-08-09

**Authors:** Mahsa Mohammadi Moqaddam, Mostafa Mirjalili, Jalil Vahdati Khaki, Sahar Mollazadeh Beidokhti

**Affiliations:** Department of Materials and Metallurgical Engineering, Faculty of Engineering, Ferdowsi University of Mashhad Mashhad 91775-1111 Iran mirjalili@um.ac.ir

## Abstract

Manganese oxides were synthesized systematically *via* the solution combustion procedure using two kinds of fuels, namely glycine and urea. The influences of the type of fuel and the fuel ratio were deeply investigated to explain the phase evolution and morphology of the product. The synthesized nanostructured powder was characterized by X-ray diffraction, particle size analysis, and FESEM. Furthermore, the thermodynamic aspects of all the synthesis reactions were studied by the calculation of the adiabatic temperature. Various manganese oxides, such as MnO, Mn_3_O_4_, Mn_2_O_3_, and MnO_2_, were obtained by varying the fuel ratio from 0.15 to 2. It was found that decreasing the fuel ratio promoted the formation of MnO_2_ by declining the combustion temperature and reductive conditions of the system. However, α-MnO_2_ could be simply achieved by adding KNO_3_ in a modified solution combustion process under fuel-lean conditions. Further heat treatment of the product was found to increase the crystallinity of the α-MnO_2_ nanoparticles.

## Abstract 2

Synthesis of α-MnO_2_ nano powders *via* a modified one-step solution combustion method. Particle size and morphology are investigated to determine how the initial composition and adiabatic temperature of the synthesis affect the phase evolution and properties of the product.

## Graphical abstract

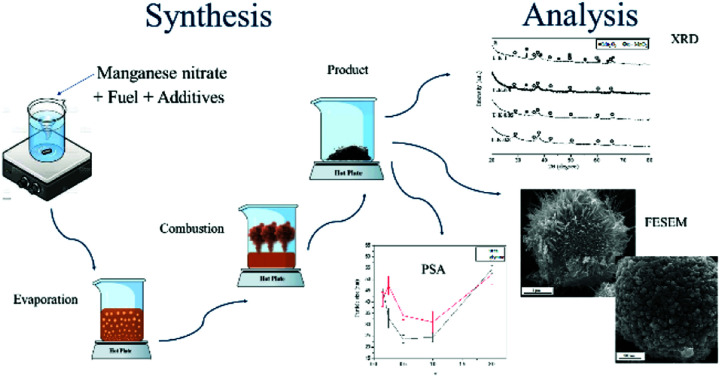

## 1 Introduction

For decades, manganese oxides (MnO, Mn_3_O_4_, Mn_2_O_3_, and MnO_2_) have been considered as promising materials due to their wide range of technological applications, including as catalysts, electrochemical materials, ion-exchange materials, and high-density magnetic storage media. Among the various types of manganese oxides, nanostructured manganese dioxide (MnO_2_) exists in various polymorph forms, such as α-, β-, γ-, δ-, ε-, and λ-MnO_2_, in which all the crystalline structures are composed of octahedral MnO_6_ building blocks,^1^ with oxygen atoms at the corners of the octahedra and Mn atoms at the center. Further, for the α-MnO_2_ structure, its double chains of edge-sharing MnO_6_ octahedra form 2 × 2 square-shaped open tunnels with dimensions of 4.6 × 4.6 Å.^2^

It has been recently reported that the catalytic activity of MnO_2_ is related to its structural features. Different polymorphs of MnO_2_ were investigated as an electrocatalyst,^3,4^ and α-MnO_2_ exhibited a great catalytic activity. A similar sequence of the ORR catalyst activity was found in MnO_2_ with different crystal forms: β-MnO_2_ < λ-MnO_2_ < γ-MnO_2_ < α-MnO_2_ ≈ δ-MnO_2_.^3^ Here, the α-MnO_2_ (Mn_8_O_16_) type often contain cations with 1^+^ or 2^+^ charges that are located within the manganese oxide tunnels where the Mn cations possess mixed 3^+^ and 4^+^ oxidation states.^5^ In general, the α-MnO_2_ oxide forms in the isostructural series with a general formula, whereby α-MnO_2_ can be formed only in the presence of a large ion, such as K^+^, and the general formula is A_2−*y*_B_8−*z*_X_16_ (A represents large ions, such as Ba^2+^, Pb^2+^ or K^+^; B is Mn^4+^, Fe^3+^, or Mn^2+^; X is O^2−^ or OH^−^; and 0.8 < *y* < 1.3 and 0.1 < *z* < 0.5). The most common form is K_*x*_Mn_8_O_16_ (cryptomelane); however, the 2 × 2 tunnels of α-MnO_2_ can reversibly host various other cations, such as Ag^+^, NH^4+^, and Na^+^.^6–8^ α-MnO_2_ has been used in lithium-ion batteries, supercapacitors, and catalysts because of its high theoretical capacitance (1370 F.g^−1^), low toxicity, low cost, natural abundance, and environmental friendliness.

Potassium-containing cryptomelane (K_*x*_Mn_8_O_16_) can be synthesized by a variety of methods, including hydrothermal^9,10^ and sol–gel^11,12^ techniques. Among these mentioned synthesis procedures, the hydrothermal method has been mostly used to synthesize α-MnO_2_. Moreover, redox sol–gel and acid digestion have been employed to synthesize K_1.2_Mn_8_O_16_ and K_1.3_Mn_8_O_16_ for electrocatalytic application, which showed S-shaped discharge curves with voltage plateaus of 3.0 V and 2.45 V.^13^ Despite their relatively high plateau voltages, they showed modest initial discharge capacities of 160 and 188 mA h g^−1^, when examined at 0.1 mA cm^−2^ and 50 mA g^−1^, respectively.^14^ In another study, cryptomelane-type tunnel-structured manganese dioxides (K_*x*_Mn_8_O_16_) with different K^+^ amounts were prepared by a hydrothermal redox reaction. The K^+^ content of K_*x*_Mn_8_O_16_ was controlled by altering the reactant ratio of K_2_SO_4_/(NH_4_)_2_SO_4_. The catalytic behavior of K_*x*_Mn_8_O_16_ with different amounts of K^+^ was evaluated using the cyclic voltammetry (CV) technique and the results suggested that K_*x*_Mn_8_O_16_ with a lower K^+^ content (*x* = 0.0, 0.32) showed a higher delivered capacity, improved capacity retention, higher discharge voltage, and higher lithium-ion diffusion coefficient (*D*_Li+_) than high K^+^ (*x* = 0.51, 0.70, 0.75)-containing K_*x*_Mn_8_O_16_.^9^ The disadvantage of these methods is that they require high temperatures, expensive precursors, and long processing times for the synthesis, which also often need special and expensive equipment.

Solution combustion synthesis (SCS) is a combustion-based process that involves converting fuel and an oxidant into a foamy crystalline material through rapid exothermic reactions. Research on SCS has attracted extensive attention in recent years, due to its various advantages, including its ability to reach high temperatures, quick heating, short reaction times, and the formation of pure products.^15–18^

In the present study, α-MnO_2_ powders were synthesized through a solution combustion synthesis method. Considering the fact that MnO_2_ is stable at low temperatures while the other forms of manganese oxide are stable at high temperatures, the synthesis of MnO_2_ was challenging by the SCS method, as this is a high-temperature process. Moreover, attaining α-MnO_2_ makes the process more complicated. Thus, various parameters, such as adiabatic temperature, adding a stabilizer, and varying the fuel/oxidizer ratio and the type of fuel, were controlled in order to produce this unique structure. More importantly, our study highlights the improvement of the physicochemical properties of the as-synthesized α-MnO_2_ powders by modifying the morphology and particle size. Furthermore, the impact of K^+^ on stabilizing α-MnO_2_ was investigated through the SCS process.

## 2 Experimental procedure

### 2.1 Synthesis of α-MnO_2_

Manganese(ii) nitrate tetrahydrate (Mn (NO_3_)_2_·4H_2_O > 99%, Merck, Germany), potassium nitrate (KNO_3_ > 99%, Merck, Germany)m and urea (CH_4_N_2_O > 99%, Neutron, Iran) were utilized as precursors.

The synthesis procedure was carried out according to the following five systems.

Stoichiometric system using glycine and urea as fuels with the ratio of the total reducing valences to total oxidizing valences being unity (*φ* = 1) for the production of MnO_2_. This system was applied to study the effect of the fuel type on the physicochemical properties of the final product (samples G-1 and U-1).

Non-stoichiometric system with various *φ* ratios (*φ* = 0.15, 0.25, 0.5, 1, and 2) using glycine: This system was used to investigate the effect of the *φ* value on the physicochemical properties of the final product (samples G-0.15, G-0.25, G-0.5, G-1, and G-2).

Non-stoichiometric system with various *φ* ratios (*φ* = 0.15, 0.25, 0.4, 0.5, 0.6, 0.8, 1, and 2) using urea: This system was used to investigate the effect of the *φ* value on the physicochemical properties of the final product (samples U-0.15, U-0.25, U-0.4, U-0.5, U-0.6, U-0.8, U-1, and U-2).

System using urea (*φ* = 0.25) with KCl as the stabilizer of α-MnO_2_ with various K/Mn ratios of 0.006, 0.030, 0.059, and 0.259: This system was used to study the effect of KCl on the physicochemical properties of the final product (samples U–KCl-0.006, U–KCl-0.030, U–KCl-0.059, and U–KCl-0.295).

System using urea with KNO_3_ as the stabilizer of α-MnO_2_ with various *φ* ratios (*φ* = 0.8, 0.85, 0.9, and 1): This system was used to investigate the effect of KNO_3_ on the physicochemical properties of the final product (samples U–K-0.8, U–K-0.85, U–K-0.9, and U–K-1). For this purpose, the mass ratio of KNO_3_ to Mn(NO_3_)_2_·4H_2_O was considered to be 0.8. The as-synthesized sample U–K-0.8 was also heat treated at 380 °C for 48 h to investigate the effect of annealing on the crystallinity of the nanoparticles (sample U–K-0.8-Ht).

Table 1 presents the balanced reactions between the nitrates, fuels, and stabilizer at various *φ* values (system 1, 2, 3, 4, and 5). In the synthesis procedure, the desired amounts of precursors were first dissolved in 10 mL of deionized water according to the equations listed in Table 1. The solution was then magnetically stirred to achieve a homogenous solution. The obtained clear solution was poured into an alumina crucible and placed on a hotplate. The solution was heated until the extra water was evaporated and a gel formed. In this step, the temperature increased and once it exceeded the ignition temperature of the reaction, combustion occurred. Then the combustion reaction was ignited in a flaming manner, resulting in the formation of crystalline nanopowders. Afterwards, the product was filtered and rinsed with deionized water several times. Finally, the powder was placed in an oven and dried at 60 °C for 1 h.

**Table 1:** Chemical reactions for the applied systems in the MnO_2_ synthesis

System	Sample code	Chemical reaction	*φ*
1	G-1	Mn(NO_3_)_2_·4H_2_O + 0.889C_2_H_5_NO_2_ = MnO_2_ + 1.778 CO_2_ (g) + 6.222H_2_O (g) + 1.444 N_2_ (g)	1
	U-1	Mn(NO_3_)_2_·4H_2_O + 1.333 CH_4_N_2_O = MnO_2_ + 1.333 CO_2_ (g) + 6.667H_2_O (g) + 2.333 N_2_ (g)	1
2	G-0.15	Mn(NO_3_)_2_·4H_2_O + 0.133C_2_H_5_NO_2_ = 0.150 MnO_2_ + 0.266 CO_2_ (g) + 0.216 N_2_ (g) + 0.934H_2_O (g) + 0.850 Mn(NO_3_)_2_·4H_2_O	0.15
	G-0.25	Mn(NO_3_)_2_·4H_2_O + 0.222C_2_H_5_NO_2_ = 0.250 MnO_2_ + 0.444 CO_2_ (g) + 1.555H_2_O (g) + 0.361 N_2_ (g) + 0.750 Mn(NO_3_)_2_·4H_2_O	0.25
	G-0.5	Mn(NO_3_)_2_·4H_2_O + 0.444C_2_H_5_NO_2_ = 0.500 MnO_2_ + 0.888 CO_2_ (g) + 3.111H_2_O (g) + 0.722 N_2_ (g) + 0.500 Mn(NO_3_)_2_·4H_2_O	0.5
	G-1	Mn(NO_3_)_2_·4H_2_O + 0.889C_2_H_5_NO_2_ = MnO_2_ + 1.778 CO_2_ (g) + 6.222H_2_O (g) + 1.444 N_2_ (g)	1
	G-2	Mn(NO_3_)_2_·4H_2_O + 1.778C_2_H_5_NO_2_ = MnO_2_ + 1.778 CO_2_ (g) + 6.222H_2_O (g) + 1.444 N_2_ (g) + 0.889C_2_H_5_NO_2_	2
3	U-0.15	Mn(NO_3_)_2_·4H_2_O + 0.200 CH_4_N_2_O = 0.150 MnO_2_ + 0.200 CO_2_ (g) + 0.350 N_2_ (g) + H_2_O (g) + 0.850 Mn(NO_3_)_2_·4H_2_O	0.15
	U-0.25	Mn(NO_3_)_2_·4H_2_O + 0.333 CH_4_N_2_O = 0.250 MnO_2_ + 0.333 CO_2_ (g) + 0.583 N_2_ (g) + 1.667H_2_O (g) + 0.750 Mn(NO_3_)_2_·4H_2_O	0.25
	U-0.4	Mn(NO_3_)_2_·4H_2_O + 0.533 CH_4_N_2_O = 0.400 MnO_2_ + 0.533 CO_2_ (g) + 2.667H_2_O (g) + 0.933 N_2_ (g) + 0.600 Mn(NO_3_)_2_·4H_2_O	0.4
	U-0.5	Mn(NO_3_)_2_·4H_2_O + 0.667 CH_4_N_2_O = 0.500 MnO_2_ + 0.667 CO_2_ (g) + 3.333H_2_O (g) + 1.166 N_2_ (g) + 0.500 Mn(NO_3_)_2_·4H_2_O	0.5
	U-0.6	Mn(NO_3_)_2_·4H_2_O + 0.800 CH_4_N_2_O = 0.600 MnO_2_ + 0.800 CO_2_ (g) + 4.000H_2_O (g) + 1.400 N_2_ (g) + 0.400 Mn(NO_3_)_2_·4H_2_O	0.6
	U-0.8	Mn(NO_3_)_2_·4H_2_O + 1.066 CH_4_N_2_O = 0.800 MnO_2_ + 1.066 CO_2_ (g) + 5.332H_2_O (g) + 1.866 N_2_ (g) + 0.200 Mn(NO_3_)_2_·4H_2_O	0.8
	U-1	Mn(NO_3_)_2_·4H_2_O + 1.333 CH_4_N_2_O = MnO_2_ + 1.333 CO_2_ (g) + 6.667H_2_O (g) + 2.333 N_2_ (g)	1
	U-2	Mn(NO_3_)_2_·4H_2_O + 2.667 CH_4_N_2_O = MnO_2_ + 1.333 CO_2_ (g) + 6.667H_2_O (g) + 2.333 N_2_ (g) + 1.333 CH_4_N_2_O	2
4	U–KCl-0.006	Mn(NO_3_)_2_·4H_2_O + 0.333 CH_4_N_2_O + 0.006 KCl = 0.250 MnO_2_ + 0.333 CO_2_ (g) + 0.583 N_2_ (g) + 1.667H_2_O (g) + 0.750 Mn(NO_3_)_2_·4H_2_O + 0.006 KCl	0.25
	U–KCl-0.030	Mn (NO_3_)_2_·4H_2_O + 0.333 CH_4_N_2_O + 0.030 KCl = 0.250 MnO_2_ + 0.333 CO_2_ (g) + 0.583 N_2_ (g) + 1.667H_2_O (g) + 0.750 Mn(NO_3_)_2_·4H_2_O + 0.030 KCl	0.25
	U–KCl-0.059	Mn(NO_3_)_2_·4H_2_O + 0.333 CH_4_N_2_O + 0.059 KCl = 0.250 MnO_2_ + 0.333 CO_2_ (g) + 0.583 N_2_ (g) + 1.667H_2_O (g) + 0.750 Mn(NO_3_)_2_·4H_2_O + 0.059 KCl	0.25
	U–KCl-0.295	Mn(NO_3_)_2_·4H_2_O + 0.333 CH_4_N_2_O + 0.295 KCl = 0.250 MnO_2_ + 0.333 CO_2_ (g) + 0.583 N_2_ (g) + 1.667H_2_O (g) + 0.750 Mn(NO_3_)_2_·4H_2_O + 0.295 KCl	0.25
5	U–K-0.8	Mn(NO_3_)_2_·4H_2_O + 2.4 CH_4_N_2_O + 2 KNO_3_ = MnO_2_ + 0.64 K_2_O + 2.4 CO_2_ (g) + 8.8H_2_O (g) + 4.04 N_2_ (g) + 0.72 KNO_3_	0.8
	U–K-0.85	Mn(NO_3_)_2_·4H_2_O + 2.55 CH_4_N_2_O + 2 KNO_3_ = MnO_2_ + 0.73 K_2_O + 2.55 CO_2_ (g) + 9.1H_2_O (g) + 4.28 N_2_ (g) + 0.54 KNO_3_	0.85
	U–K-0.9	Mn(NO_3_)_2_·4H_2_O + 2.7 CH_4_N_2_O + 2 KNO_3_ = MnO_2_ + 0.82 K_2_O + 2.7 CO_2_ (g) + 9.4H_2_O (g) + 4.52 N_2_ (g) + 0.36 KNO_3_	0.9
	U–K-1	Mn(NO_3_)_2_·4H_2_O + 3 CH_4_N_2_O + 2 KNO_3_ = MnO_2_ + K_2_O + 3 CO_2_ (g) + 10H_2_O (g) + 3.4 N_2_ (g)	1

### 2.2 Characterization

The phase composition of the synthesized samples was analyzed using an Explorer GNR X-ray diffraction (XRD) system using Cu-K_α_ radiation (*λ* = 0.15418 nm) with step-scanning over the range of 12° to 80° and a step size of 0.02°. All the XRD patterns were investigated by X'Pert High Score Plus software. The particle-size distribution of combusted powders was estimated using a Cordonuan Vasco3 particle-size analyzer (PSA). The morphology and microstructure of the optimum samples were investigated on a Tescan Brno-Mira3 LMU field-emission scanning electron microscopy (FE-SEM) system.

## 3 Results and discussion

### 3.1 Thermodynamic aspects

**Fig. 1:**
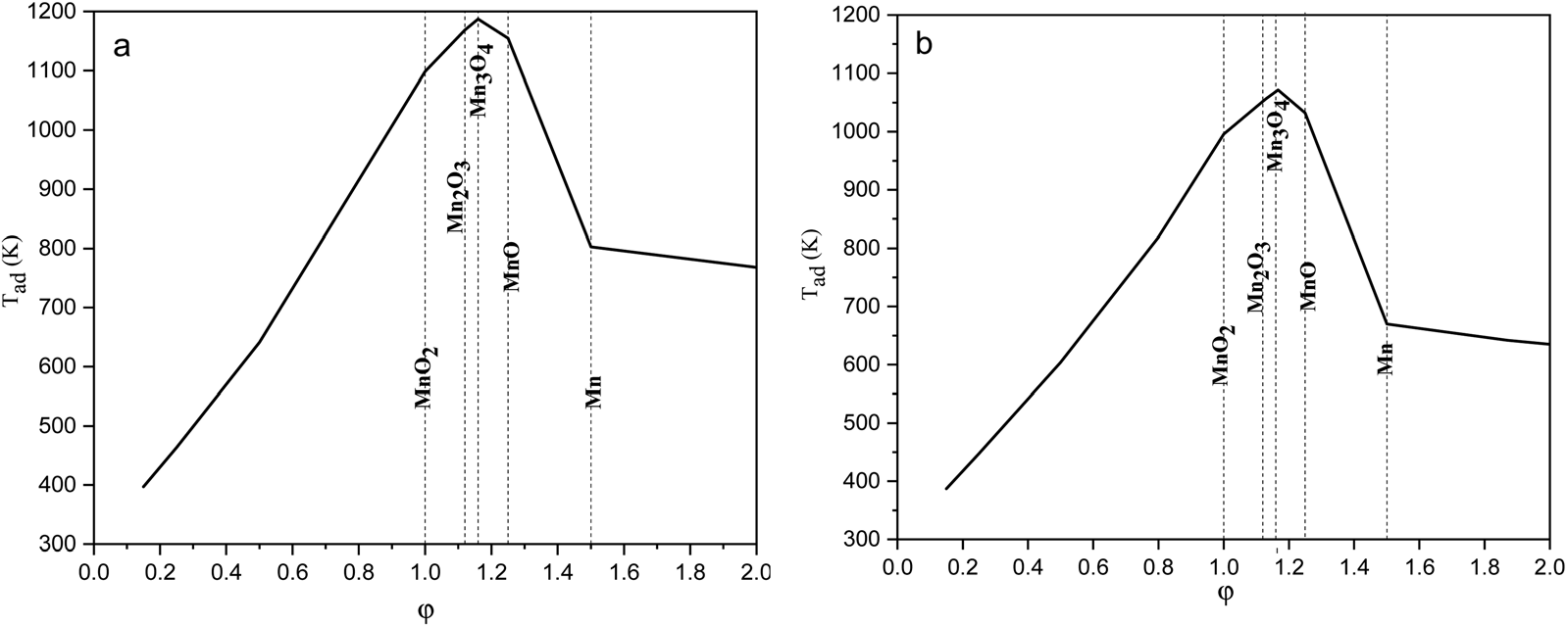
Adiabatic temperatures of the reactions *versus* the *ϕ* value considering (a) glycine, and (b) urea as fuel.

The adiabatic temperature, *T*_ad_, of all the experimental systems was calculated on the basis of eqn (1) (ref. 17) by using HSC Chemistry 6.0 software. It should be noted that all the calculations were applied for Mn(NO_3_)_2_·6H_2_O, since the thermodynamic data for Mn(NO_3_)_2_·4H_2_O, which was used as the precursor in our experiments, were not available.1
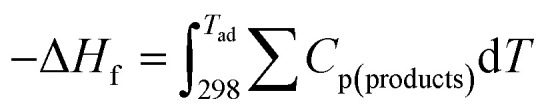
where *C*_p_ and Δ*H*_f_^0^ are the molar heat capacity at constant pressure and standard enthalpy of the reaction, respectively. Fig. 1 indicates the dependency of the adiabatic temperature on the *φ* values for glycine and urea fuels. The adiabatic temperature for the stoichiometric reactions of MnO_2_, Mn_2_O_3_, Mn_3_O_4_, MnO, and Mn are illustrated by dashed lines. Accordingly, the region between two lines shows the *φ* range for the stability of the mixed products, which are labeled on both lines. Obviously, for *φ* values less than 1.16, there was an excess amount of manganese nitrate that remained in product and acted as a diluent to reduce the adiabatic temperature. Similarly, for *φ* values more than 1.5, some fuel remained in the product and decreased the adiabatic temperature. As indicated in Fig. 1, the adiabatic temperature decreased with deviating the *φ* ratio from the value related to the production of Mn_3_O_4_ (*φ* = 1.16). Comparing Fig. 1(a) and (b) shows that the adiabatic temperature of the reactions was generally lower when urea was used as the fuel.

**Fig. 2:**
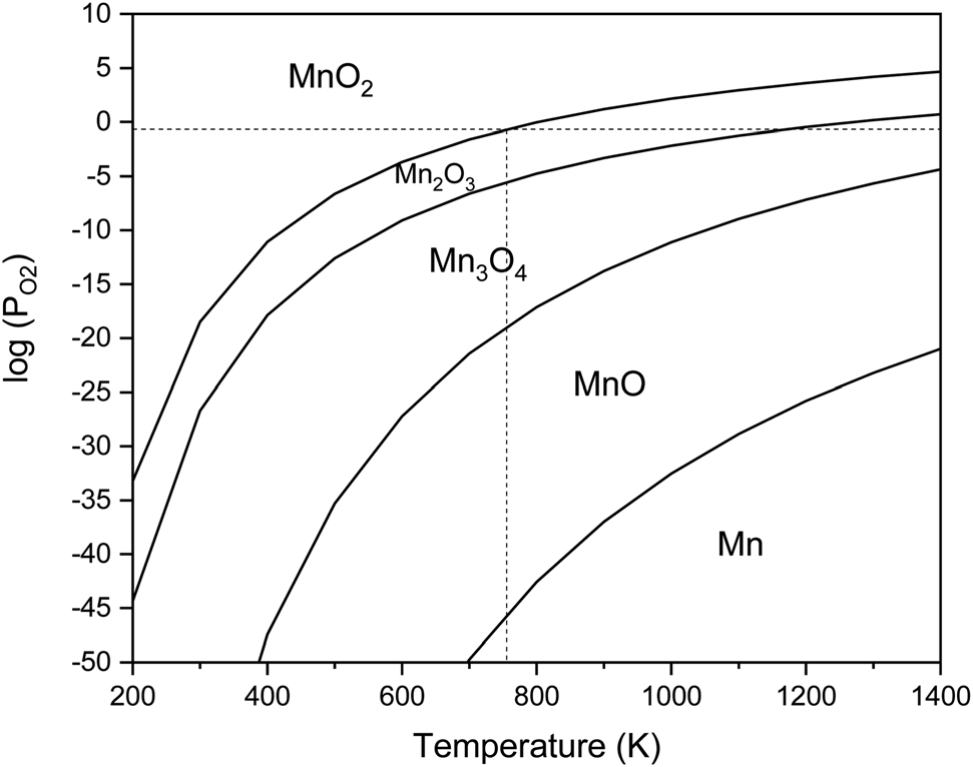
Stability regions of MnO_2_, Mn_2_O_3_, Mn_3_O_4_, MnO, and Mn in terms of the temperature and oxygen pressure.

The equilibrium oxygen pressure ranges for the stability of MnO_2_, Mn_2_O_3_, Mn_3_O_4_, MnO, and Mn are shown *versus* the temperature in Fig. 2, which were calculated by HSC Chemistry software. As indicated, at ambient atmosphere (*P*_O_2__ = 0.21atm), MnO_2_ was stable until the temperature reached 755 K; while the stability temperature ranges of Mn_2_O_3_ and Mn_3_O_4_ were 755–1172 K and 1172–1853 K, respectively. Accordingly, the enhanced temperatures obtained during the synthesis were not favorable for the production of MnO_2_. Thus, *φ* values less than 1, which lead to lower adiabatic temperatures, are more desirable for the synthesis of MnO_2_.

### 3.2 X-Ray diffraction results

**Fig. 3:**
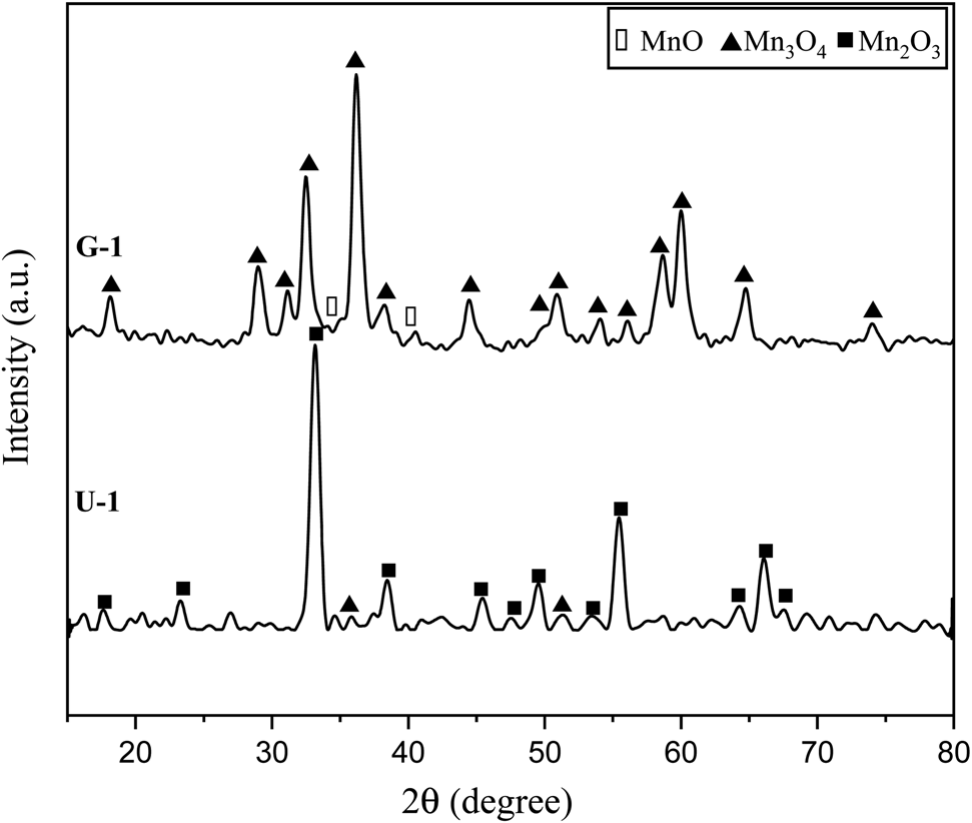
XRD patterns of the synthesized powders in the stoichiometric system using glycine and urea with *φ* = 1.

Fig. 3 presents the XRD patterns of the synthesized powders according to the reactions mentioned for system 1 in Table 1 (samples G-1 and U-1). The purpose of this system was to assess the impact of the fuel type (glycine and urea) on the physicochemical properties of the synthesized manganese oxides. As indicated in Fig. 3, sample G-1 dominantly consisted of Mn_3_O_4_ (ICDD 24-0734) with traces of MnO (ICDD 075-0626); whereas, sample U-1 mostly showed Mn_3_O_4_ with insignificant amounts of Mn_2_O_3_ (ICDD 041-1442). In contrast with the theoretical expectations for system 1 (Table 1), MnO_2_ was not formed in these samples. This may be due to the decomposition of manganese nitrate, which is probable from the low temperatures. Therefore, a part of the manganese nitrate may have been decomposed during heating and combustion, which would have disturbed the initial stoichiometric ratio of the fuel and nitrate. Thus, there was an excess amount of fuel, which caused a more reductive condition and consequently led to the formation of Mn_2_O_3_, Mn_3_O_4_, and MnO instead of MnO_2_. Moreover, since the combustion temperature was higher than the stability temperature of MnO_2_ (which was less than 755 K, according to Fig. 2), the likely formed MnO_2_ particles could deform to give the oxides with lower valences.

Comparison of the results obtained by glycine and urea showed that glycine caused a more reductive condition, yielding the formation of Mn_3_O_4_ and MnO, while in the synthesis by urea, Mn_3_O_4_ and Mn_2_O_3_ were formed. This can be attributed to the lower boiling point of urea (135 °C) rather than glycine (241 °C). Therefore, during the heating up till the ignition of the synthesis, greater amounts of urea can probably evaporate; thus, the synthesis condition by glycine seems to be more reductive in the empirical condition. Moreover, the higher adiabatic temperature of the synthesis by glycine provides the proper condition for stability of the oxides with lower valences, as discussed in Fig. 2. Besides, it is obvious that the synthesis by glycine produced a lesser amount of gases (Table 1, system 1), which reduces the heat loss and consequently decreases the combustion temperature drop. Therefore, it could be concluded that urea is a better fuel for the synthesis of MnO_2_ due to its lower combustion temperature.

**Fig. 4:**
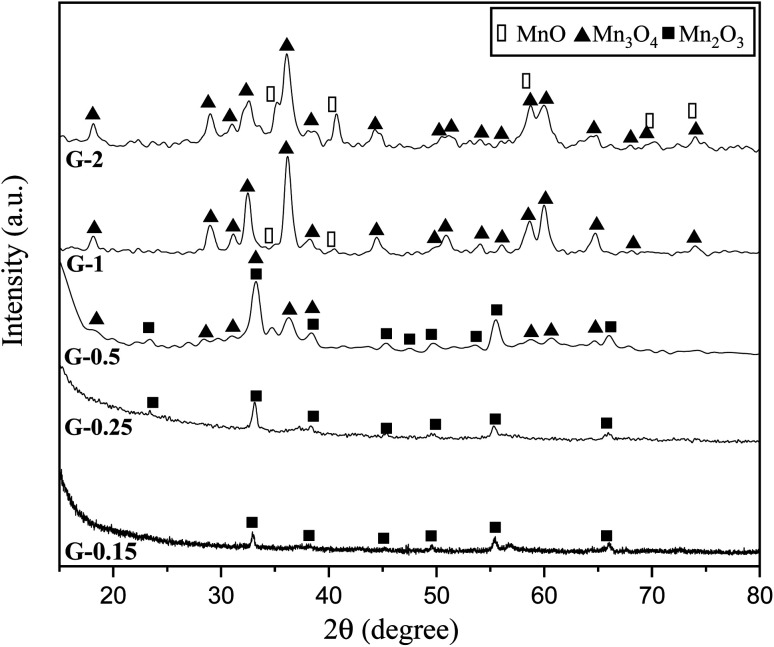
XRD patterns of the synthesized powders in the non-stoichiometric system with various *φ* ratios (*φ* = 0.15, 0.25, 0.5, 1, and 2) using glycine.

Fig. 4 indicates the XRD patterns of the samples of system 2. In this system, non-stoichiometric reactions with various *φ* ratios (*φ* = 0.15, 0.25, 0.5, 1, and 2) using glycine were assessed to study the effect of the *φ* value on the types of produced manganese oxides (samples G-0.15, G-0.25, G-0.5, G-1, and G-2). As can be seen, sample G-2 consisted of Mn_3_O_4_ and MnO oxides.^19^ However, the thermodynamic calculations shown in Fig. 1 suggest that metallic Mn could be expected at *φ* = 2. This can be attributed to the fact that the stability temperature of Mn, which is indicated in Fig. 2, is higher than the adiabatic temperature of the system. Although, if some Mn is formed, it would be oxidized by the oxygen in the atmosphere during the synthesis.

According to Fig. 1, it could be anticipated that MnO_2_ would form in the samples with *φ* = 1 and less (*i.e.*, samples G-0.15, G-0.25, G-0.5, and G-1). However, the XRD patterns showed Mn_3_O_4_ as the dominant oxide in sample G-1, and Mn_2_O_3_ as the dominant phase in samples G-0.15, G-0.25, and G-0.5. This could be attributed to the decomposition of manganese nitrate during heating and synthesis which increased the fuel to oxidizer ratio and provided a more reductive condition. As shown in Fig. 4, decreasing the *φ* value from 1 to 0.15 gradually led to the formation of Mn_2_O_3_ due to less reductive condition and the lower adiabatic temperature of the system. Despite the low *φ* value and adiabatic temperature, which provide proper conditions for the formation of MnO_2_, no evidence of MnO_2_ was observed in the XRD patterns, even for sample G-0.15. Accordingly, further attempts were followed using urea as a fuel (systems 3, 4, and 5), as described in Table 1.

**Fig. 5:**
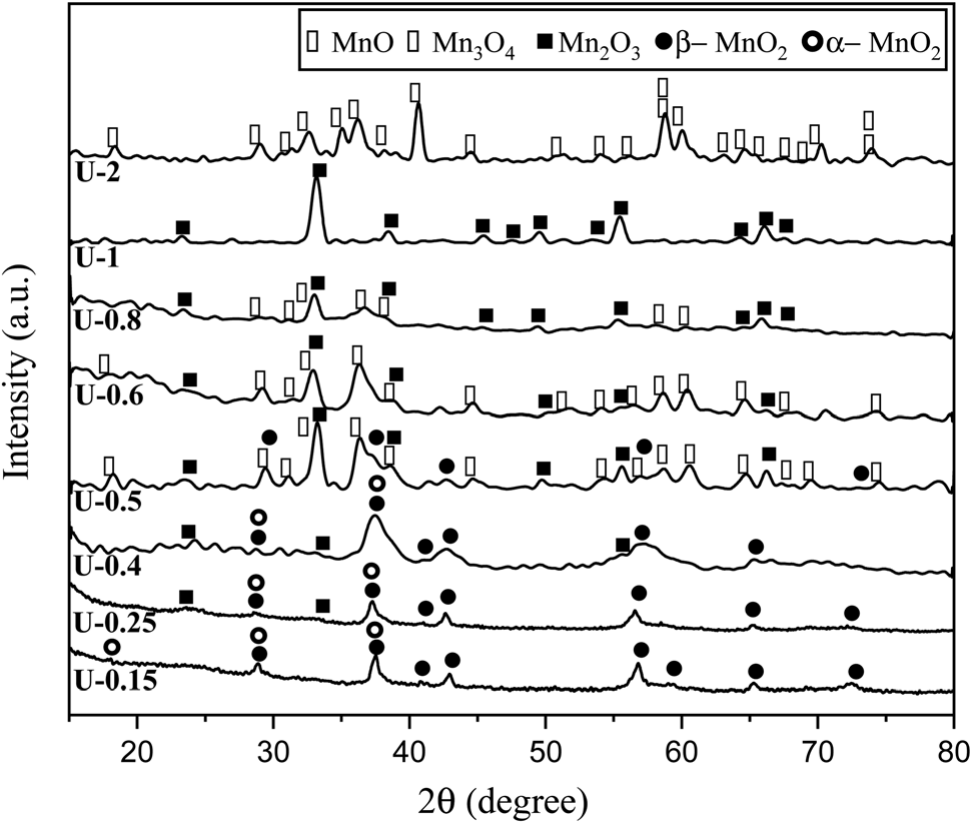
XRD patterns of the synthesized powders in the non-stoichiometric system with various *φ* ratios (*φ* = 0.15, 0.25,0.4, 0.5, 0.6, 0.8, 1, and 2) using urea.

The XRD patterns of the products attained by system 3 are shown in Fig. 5. In this system non-stoichiometric reactions with various *φ* ratios (*φ* = 0.15, 0.25, 0.4, 0.5, 0.6, 0.8, 1, and 2) using urea were investigated (samples U-0.15, U-0.25, U-0.4, U-0.5, U-0.6, U-0.8, U-1, and U-2). As indicated, sample U-2 did not show Mn formation because the temperature during the synthesis did not reach the stability temperature of Mn. This sample was mostly formed of Mn_3_O_4_ and MnO oxides.^19^

The XRD pattern of sample U-1 showed Mn_2_O_3_ as the dominant oxide. However, MnO_2_ was expected to form at *φ* = 1 and less. It returned to the decomposition of manganese nitrate during heating, which increases the real fuel to oxidizer ratio, resulting in a more reductive condition. Besides, the high combustion temperature of this sample was not appropriate for the formation of MnO_2_ which is stable at low temperatures.

Although it was anticipated that manganese oxides with higher valences, such as MnO_2_ and Mn_2_O_3_, would form with decreasing the *φ* ratio to values less than 1, an abnormal behavior was observed in the samples U-0.5, U-0.6, and U-0.8, which revealed Mn_3_O_4_. This could be attributed to the dissimilar conditions of urea evaporation during the heating and gel formation, which led to the unexpected behavior. This behavior was not observed in the samples synthesized by glycine because of the higher boiling point of glycine.

**Fig. 6:**
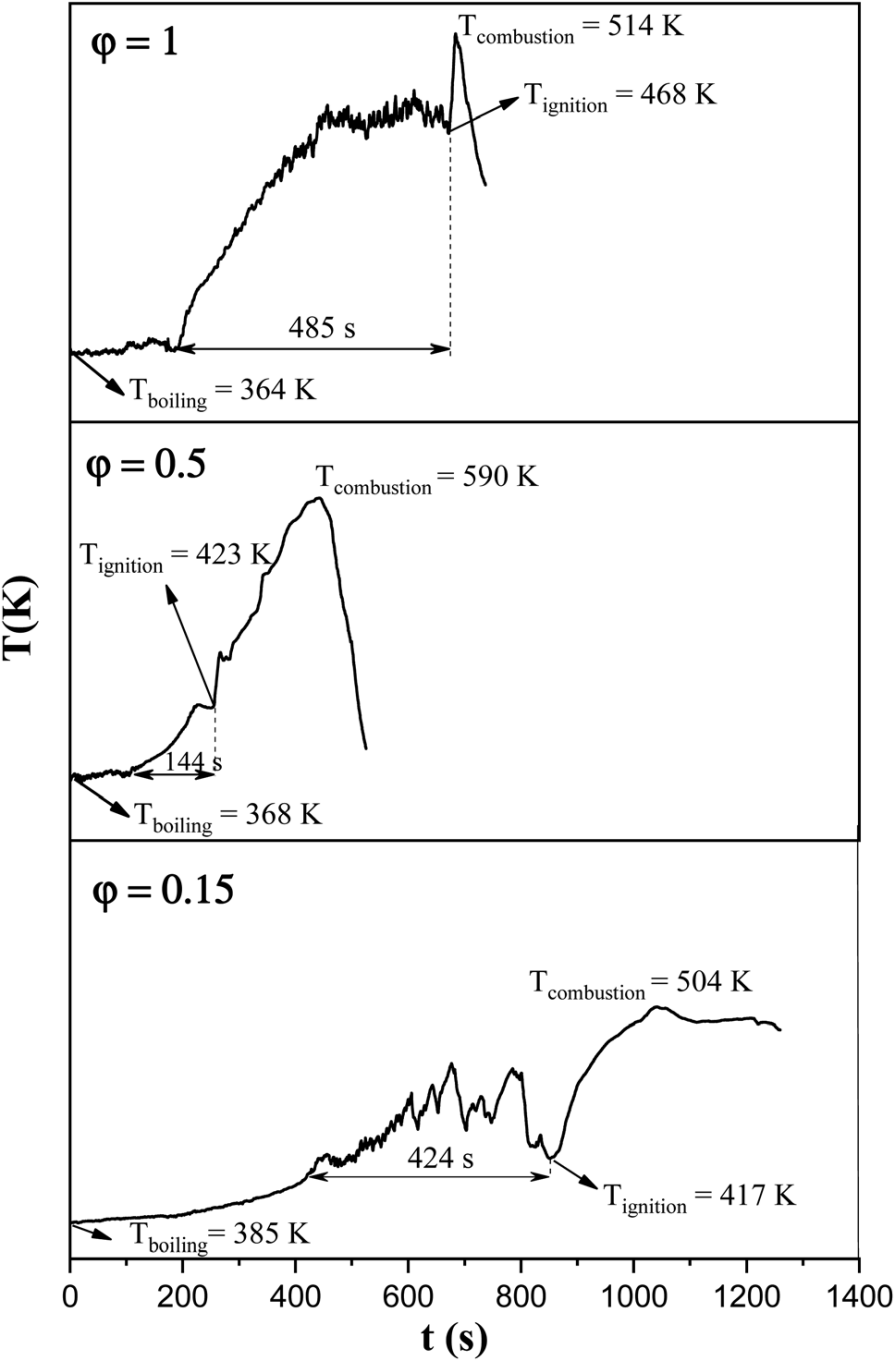
Temperature–time profile during the synthesis of the samples U-0.15, U-0.5, and U-1.

In order to assess the impact of the *φ* value on the temperature and duration of the pre-combustion step, the temperature was measured and plotted *versus* time during the synthesis of the third system. Fig. 6 displays the thermal profiles of the samples U-0.15, U-0.5, and U-1, which were obtained using a K-type thermocouple inserted in the synthesis environment and a data acquisition system for recording the temperature with time. A schematic of the main steps is illustrated in Table 2, including boiling, pre-combustion step (gel formation), combustion, and cooling. In the first step, the solution is heated to reach boiling point. During the boiling step, the temperature remains constant. Concentration of the solution gradually increases, leading to gel formation (pre-combustion step), which is accompanied by an enhancement of the temperature. Since the evaporation of fuel occurs rapidly in the pre-combustion step, the duration of this step has a considerable effect on the real *φ* value and reductive condition of the system. Elongation of this step yields more fuel loss and a less reductive condition. At the end of this step, ignition occurs, which is followed by smoldering. Finally, the products form and cool down to room temperature.

**Table 2:** Schematics and top views of the main steps of the solution combustion synthesis process

Boiling step	Pre-combustion step (gel formation)	Combustion	Cooling
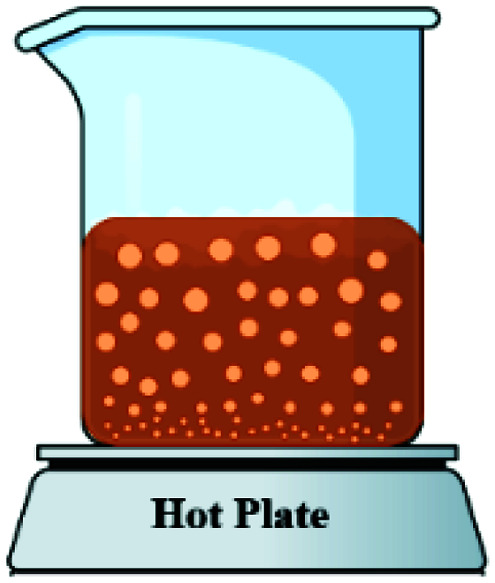	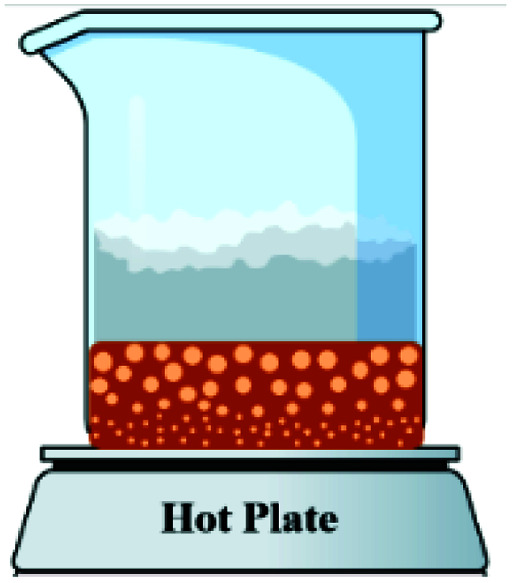	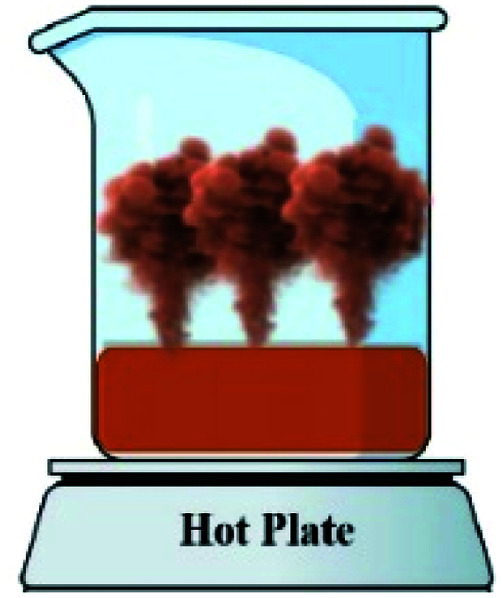	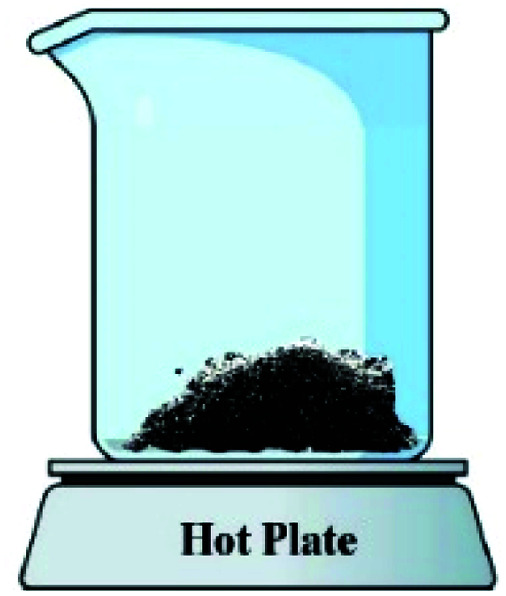
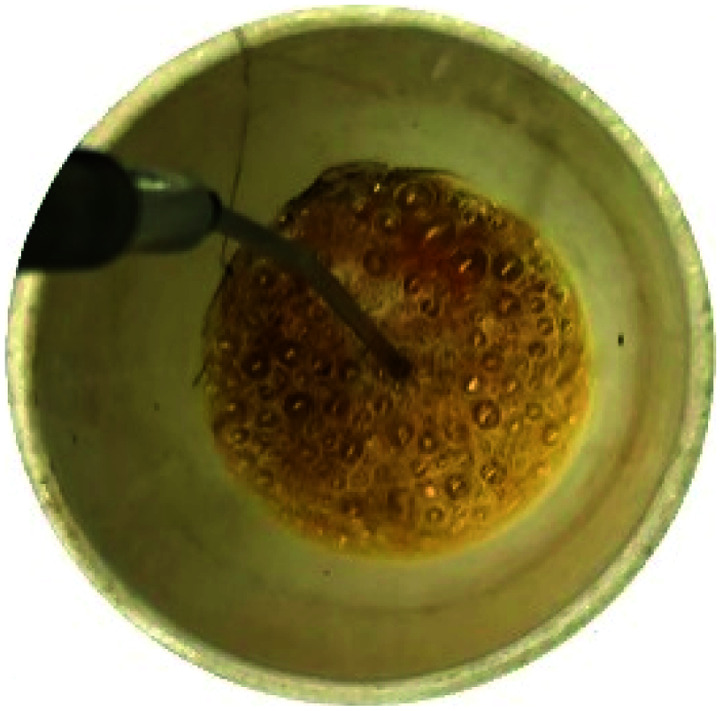	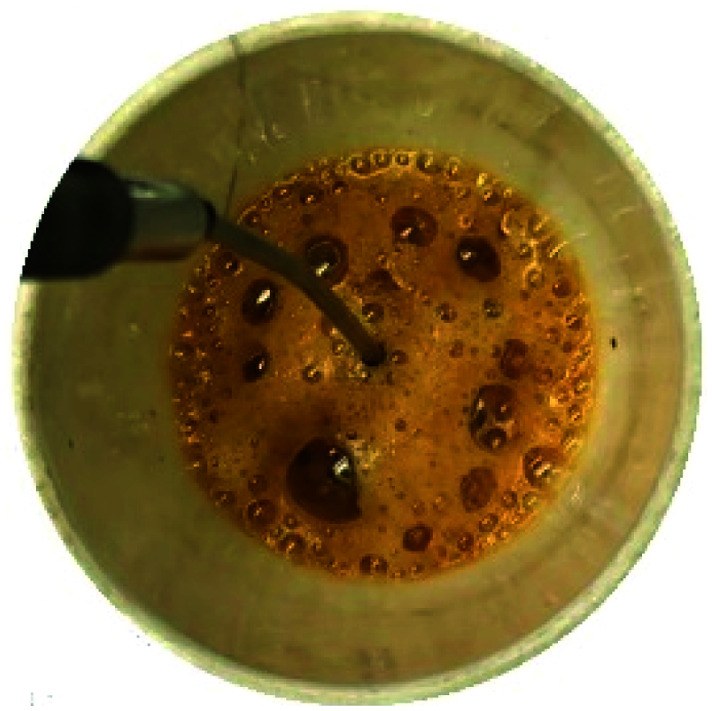	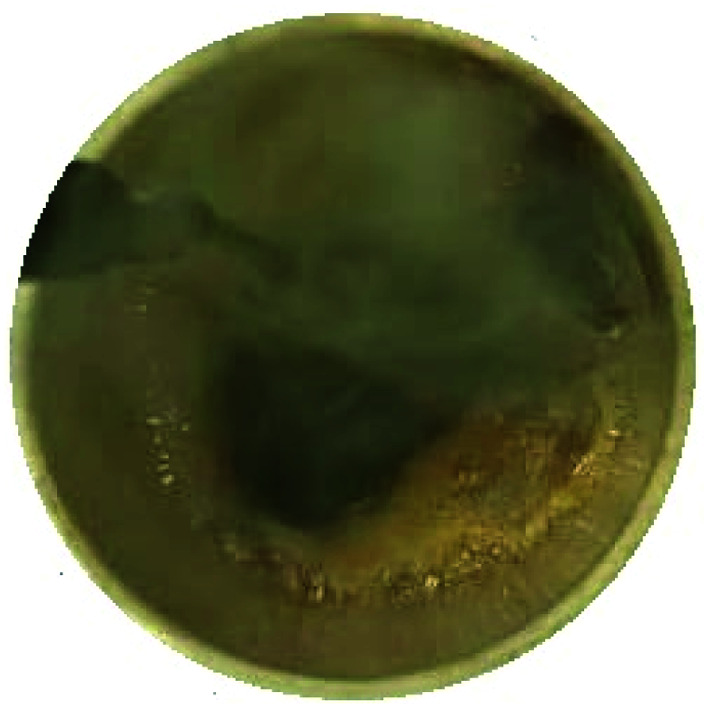	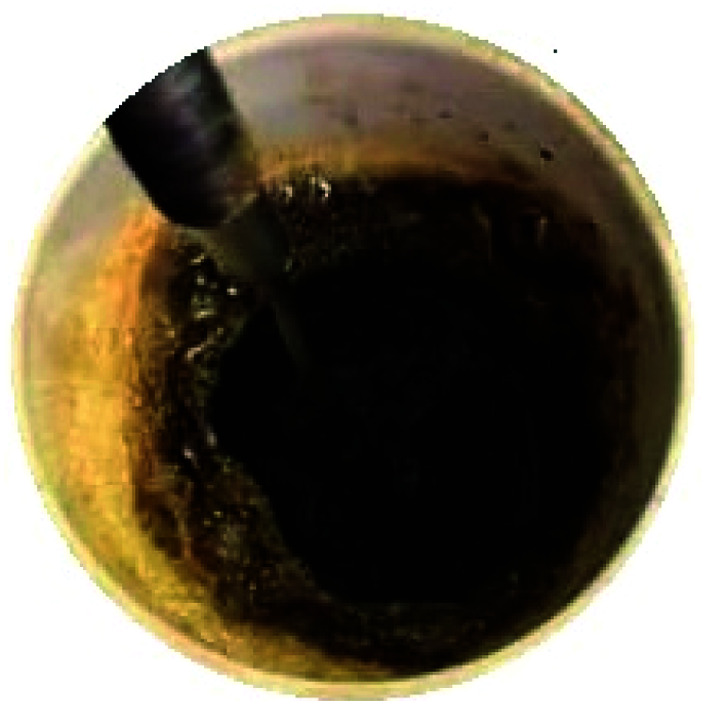

As depicted in Fig. 6, at *φ* = 0.5 (sample U-0.5), the pre-combustion time and the ignition temperature, which represents the final temperature of the pre-combustion step, were both lower than for the sample with *φ* = 1 (sample U-1). Thus, less urea evaporation occurred in sample U-0.5, resulting in a more reductive environment. Therefore, as shown in Fig. 5, Mn_3_O_4_ would be more likely to form at *φ* = 0.5 compared to at *φ* = 1. Accordingly, the unexpected formation of Mn_3_O_4_ by reducing the *φ* value from 1 to 0.8, 0.6, and 0.5 can be explained by the different pre-combustion duration and temperature.

Further decreasing the *φ* value (*e.g.*, *φ* = 0.15) led to a prolonged pre-combustion time, as demonstrated in Fig. 6, which caused a higher loss of urea and thus a more oxidizing environment, which yielded manganese oxides with higher valences. Returning to Fig. 5, it could be found that reducing the *φ* value from 0.5 to 0.4, 0.25, and 0.15, which declined the reductive condition and the adiabatic temperature of the system, gradually led to the formation of oxides with higher valances, such as Mn_2_O_3_ and MnO_2_. Accordingly, in samples U-0.15 and U-0.25, the predominant amount of MnO_2_ was formed in two types: β-MnO_2_ (ICDD 24-0735) and α-MnO_2_ (ICDD 44-0141) polymorphs. However, the formation of α-MnO_2_ in these samples was insignificant and most of the MnO_2_ was revealed to be in the β structure.

**Fig. 7:**
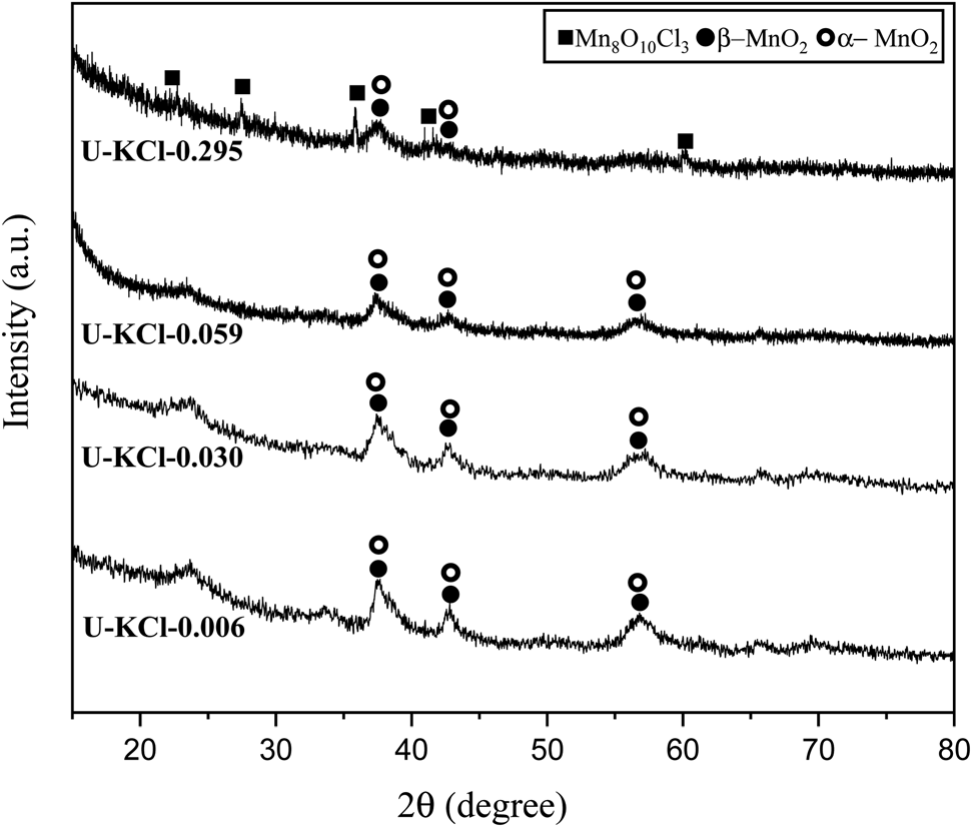
XRD patterns of the synthesized powders in the system using urea (*φ* = 0.25) with the addition of KCl in various K/Mn ratios of 0.006, 0.030, 0.059, and 0.259.

It was previously found that cations such as K^+^ can stabilize the structure of α-MnO_2_.^5,20^ Consequently, KCl was added to the reaction mixture as a source of K^+^ in various K/Mn ratios of 0.006, 0.030, 0.059, and 0.259. Referring to Table 1, system 4, four samples, namely U–KCl-0.006, U–KCl-0.030, U–KCl-0.059, and U–KCl-0.259, were synthesized, and their XRD patterns are indicated in Fig. 7. As shown, the crystallinity of the samples was low and wide peaks appeared. Thus, the phase detection was carried out based only on the major peaks. As can be seen, a mixture of α and β-MnO_2_ was formed in the presence of K^+^; however, extra amounts of KCl decreased the intensity of MnO_2_ peaks and led to the formation of MnO_10_Cl_8_ (ICDD 30-0821). It could be noted that adding KCl did not have any significant effect on the adiabatic temperature.

**Fig. 8:**
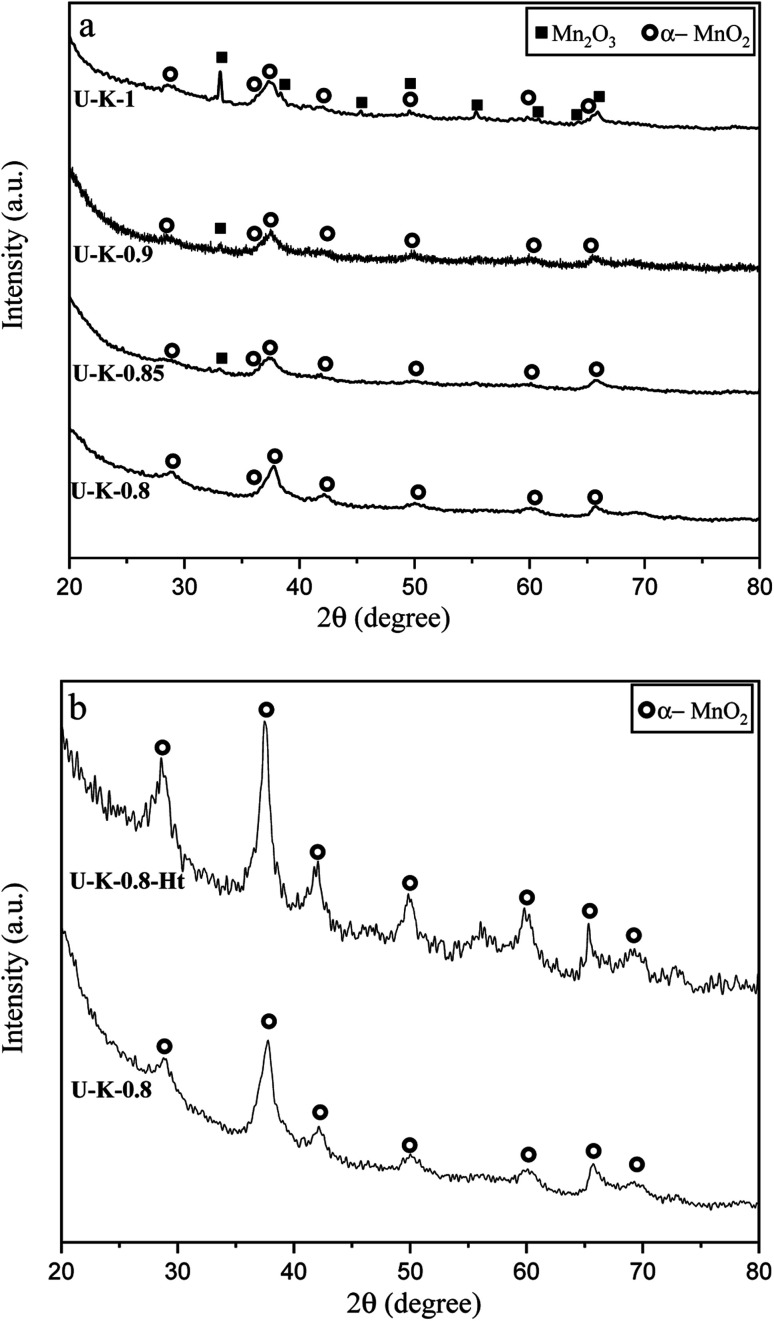
XRD patterns of the synthesized powders in (a) the system using urea with the addition of KNO_3_ in various *φ* ratios (*φ* = 0.8, 0.85, 0.9, and 1), (b) the sample synthesized at *φ* = 0.8 and annealed at 380 °C for 48 h.

Fig. 8(a) presents the XRD patterns related to the specimens synthesized with KNO_3_ as the stabilizer of α-MnO_2_ in various *φ* ratios (*φ* = 0.8, 0.85, 0.9, and 1). This system was investigated to study the effect of KNO_3_ on the formation of α-MnO_2_ in the final product (samples U–K-0.8, U–K-0.85, U–K-0.9, and U–K-1). As indicated, sample U–K-1 mostly consisted of α-MnO_2_ with some amounts of Mn_2_O_3_. As can be seen, the intensity of the peaks related to the Mn_2_O_3_ phase gradually decreased with reducing the *ϕ* value; until in sample U–K-0.8, an almost single phase of α-MnO_2_ was formed. The broadened peaks of α-MnO_2_ were a sign of the fine particle size of the synthesized powder. The stability of α-MnO_2_ could be attributed to the presence of K^+^ and the less reductive condition provided by the low *φ* values. The calculated adiabatic temperature of the reactions related to samples U–K-0.8, U–K-0.85, U–K-0.9, and U–K-1 were 923, 945, 966, and 1008 K, respectively. These values were higher than the relevant adiabatic temperatures calculated for the specimens without KNO_3_ (Fig. 1(b)), which was not proper for α-MnO_2_ formation. However, the influence of K^+^ overcame this and resulted in the stability of α-MnO_2_.

The as-synthesized sample U–K-0.8 was then annealed at 380 °C for 48 h to improve the crystallinity of the nanoparticles (sample U–K-0.8-Ht). As shown in Fig. 8(b), the intensity of the peaks increased due to the heat treatment, which implied a higher crystallinity of the sample.

### 3.3 Results of the particle-size analysis

**Fig. 9:**
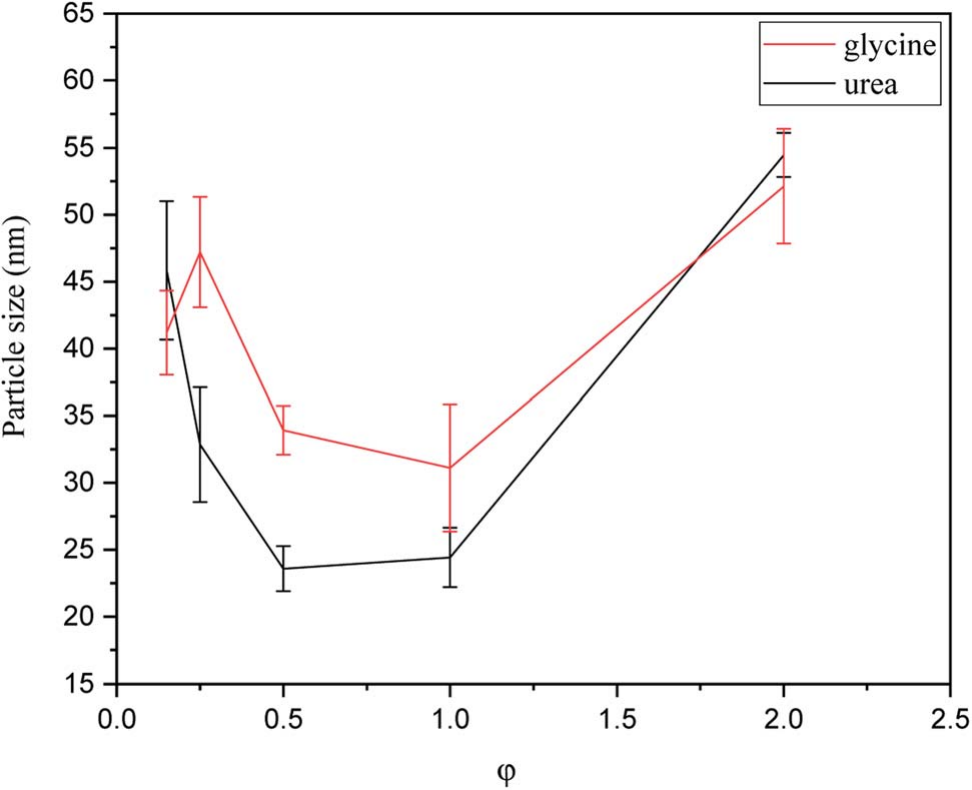
PSA results of the powders synthesized by glycine and urea in various *φ* ratios.

In order to investigate the particle size of synthesized powders, PSA was carried out and the averaged results are shown in Fig. 9. As indicated, the samples synthesized by urea had smaller particle sizes than glycine. An explanation for this phenomenon may be related to the greater volume of combustion gas produced during the synthesis with urea. According to Table 1 (system 1), 9.444 mol of combustion gases was released during the synthesis with glycine; however, this amount for the synthesis with urea was 10.333 mol. Thus, a greater dispersion of powder particles occurred when urea was used, which resulted in a reduction of the possibility of the particles adhering and agglomeration.

As can be seen in Fig. 9, the particle size also varied by changing the *φ* value. Among the samples synthesized by glycine, the sample with *φ* = 1 (G-1) showed the minimum particle size. As discussed in Fig. 4, sample G-1 was mostly composed of Mn_3_O_4_, which was accompanied with the highest combustion temperature. When the combustion temperature was high, the rate of combustion increased, causing the products to form more quickly and not to stay at the enhanced temperatures for a long time. Therefore, the sintering and agglomeration of the particles were decreased in sample G-1 and consequently a finer particle size was achieved. Comparison of the samples synthesized by urea in various *φ* values showed that the minimum particle size was attained at *φ* = 0.5–1. Referring to Fig. 5, these samples mostly consisted of Mn_3_O_4_ and Mn_2_O_3_. Accordingly, the highest combustion temperature and rate occurred in the samples yielding the smallest particle size.

### 3.4 Field-emission scanning electron microscopy (FESEM)

**Fig. 10:**
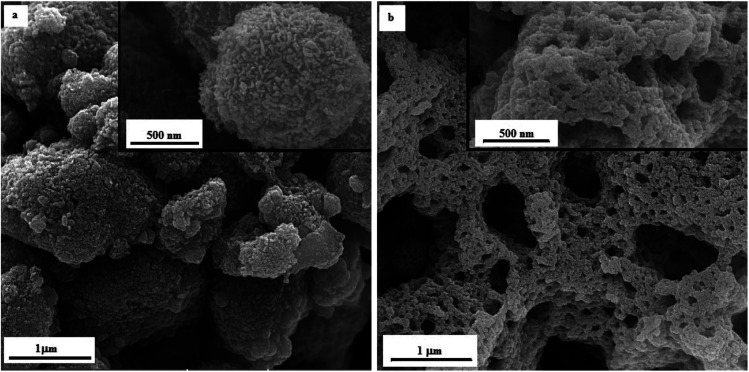
FESEM micrographs of the as-synthesized samples (a) G-0.25, and (b) G-1.

As proof of evidence, FESEM imaging was carried out to reveal the morphology of the as-synthesized manganese oxides obtained by glycine and urea at various *φ* ratios. Fig. 10 indicates the morphology of the samples G-0.25 and G-1 in different magnifications. As shown in Fig. 10(a), sample G-0.25 revealed a granular morphology, indicating agglomerates of Mn_2_O_3_ nanoparticles with a ridged surface. However, sample G-1 (Fig. 10(b)) displayed a lacy morphology. Increasing the *φ* value led to the formation of Mn_3_O_4_ and MnO nanoparticles and consequently a higher combustion temperature, resulting in more agglomeration and sintering of the nanoparticles. On the other hand, the amounts of released gases were higher in sample G-1, which obviously caused greater porosity and more cavities.

**Fig. 11:**
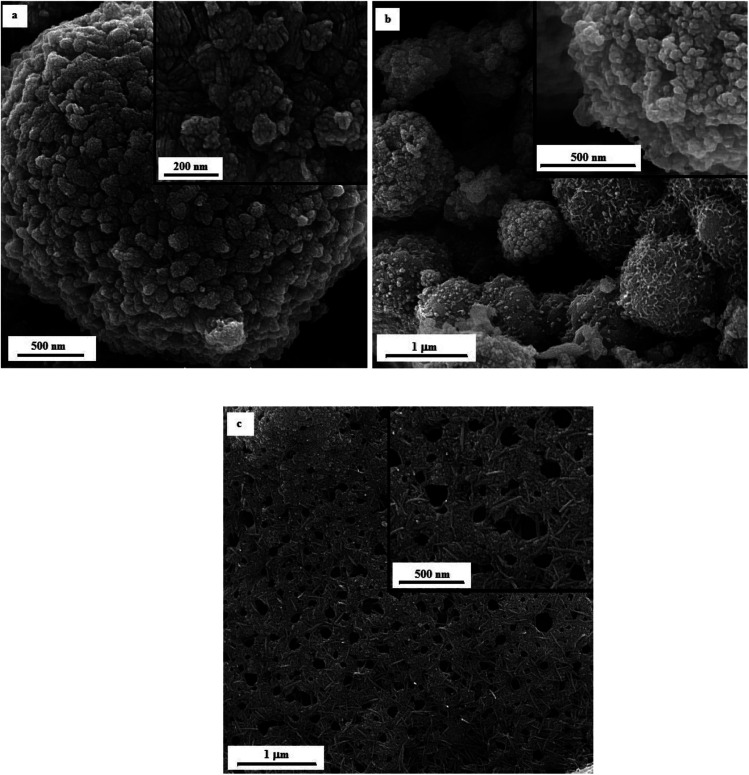
FESEM micrographs of the as-synthesized samples: (a) U-0.15, (b) U-1, and (c) U-2.

The FESEM image of sample U-0.15 is presented in Fig. 11(a), which shows the MnO_2_ nanoparticles had a cauliflower-like morphology. However, sample U-1 (Fig. 11(b)) exhibited a granular morphology for Mn_2_O_3_ nanoparticles, as also revealed for sample G-0.25. Increasing the *φ* value to 2 (*i.e.*, sample U-2) led to a lacy network morphology, as demonstrated in Fig. 11(c). Similar to sample G-1, this morphology can be attributed to the sintering of Mn_3_O_4_ and MnO nanoparticles, which was accompanied by the formation of cavities due to the huge volume of released gases.

**Fig. 12:**
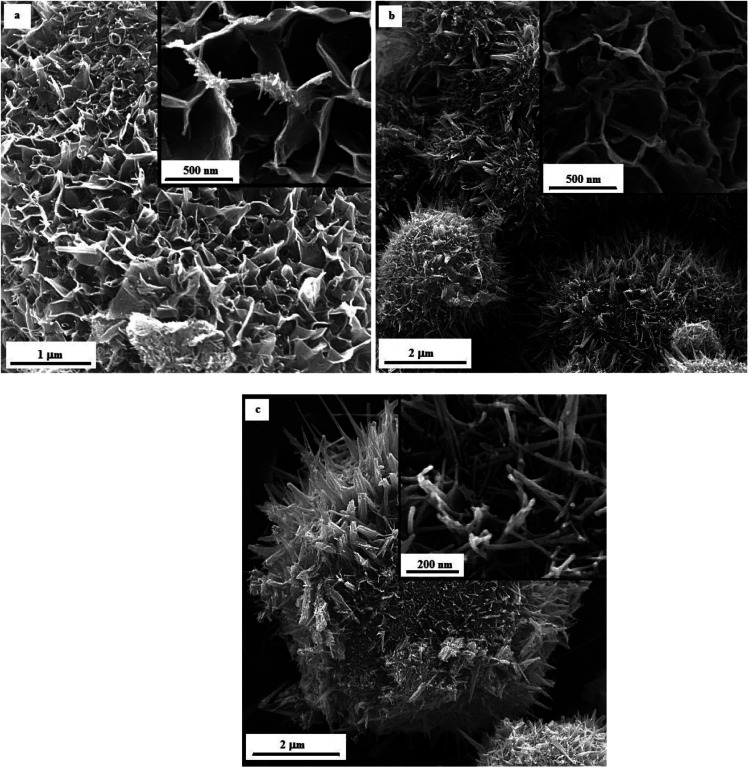
FESEM images of the as-synthesized samples: (a) U–K-0.8, (b) U–K-0.9, and (c) the heat-treated sample U–K-0.8-Ht.

Fig. 12 illustrates the FESEM micrographs of the samples synthesized by adding KNO_3_. A dandelion-like morphology with a needle/flake-like surface was revealed for the α-MnO_2_ nanoparticles in these samples. Increasing the *φ* value from 0.8 (sample U–K-0.8) to 0.9 (sample U–K-0.9) led to a finer morphology. Heat treating the product at 380 °C for 48 h resulted in fracture of the surface needles and ridges due to recrystallization and/or thermal stresses.

## 4 Conclusion

A new approach for the production of α-MnO_2_ nanoparticles was achieved using a modified solution combustion synthesis (SCS), in which α-MnO_2_ was synthesized in a one-step procedure with the addition of a stabilizer. To examine the effects of the fuel type, stabilizer, and fuel ratio on the morphology and phase composition of the final product, combustion synthesis was conducted using glycine and urea as the fuel and KCl and KNO_3_ as the stabilizer. The production of single-phase α-MnO_2_ was attained using urea under lean-fuel conditions (*i.e.*, *φ* = 0.8) and in the presence of KNO_3_ as the stabilizer. The synthesized α-MnO_2_ exhibited a specific nanostructured dandelion-like morphology. Heat treating the synthesized α-MnO_2_ at 380 °C led to greater crystallinity.

## Conflicts of interest

There are no conflicts to declare.

## Supplementary Material
